# Ewald: an extended wide-angle Laue diffractometer for the second target station of the Spallation Neutron Source

**DOI:** 10.1107/S1600576717010032

**Published:** 2017-07-26

**Authors:** Leighton Coates, Lee Robertson

**Affiliations:** a Oak Ridge National Laboratory, 1 Bethel Valley Road, Oak Ridge, TN 37831, USA

**Keywords:** neutron diffraction, protein crystallography

## Abstract

Visualizing hydrogen atoms in biological materials is one of the biggest remaining challenges in biophysical analysis. While X-ray techniques have unrivaled capacity for high-throughput structure determination, neutron diffraction is uniquely sensitive to hydrogen atom positions in crystals of biological materials and can provide a more complete picture of the atomic and electronic structures of biological macromolecules. This information can be essential in providing predictive understanding and engineering control of key biological processes, for example, in catalysis, ligand binding and light harvesting, and to guide bioengineering of enzymes and drug design. One very common and large capability gap for all neutron atomic resolution single-crystal diffractometers is the weak flux of available neutron beams, which results in limited signal-to-noise ratios giving a requirement for sample volumes of at least 0.1 mm^3^. The ability to operate on crystals an order of magnitude smaller (0.01 mm^3^) will open up new and more complex systems to studies with neutrons which will help in our understanding of enzyme mechanisms and enable us to improve drugs against multi resistant bacteria. With this is mind, an extended wide-angle Laue diffractometer, ‘Ewald’, has been designed, which can collect data using crystal volumes below 0.01 mm^3^.

## Abstract 2

The design of a macromolecular neutron diffractometer for the second target station of the Spallation Neutron Source is detailed.

## 1. Introduction   

**Figure 1:**
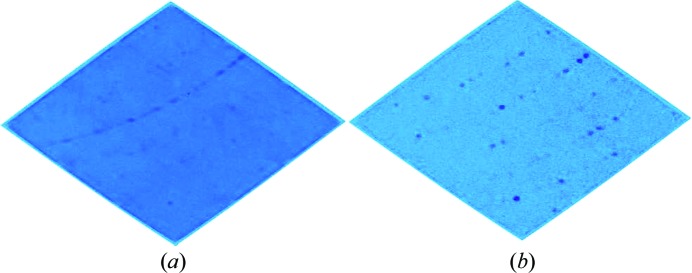
(*a*) A Laue diffraction image from a single SNS Anger camera recorded on the MaNDi instrument using all wavelengths between 2 and 4.16 Å. (*b*) A diffraction pattern from the same detector, this time looking only at neutrons between 3.0 and 3.1 Å. As can be seen, the time of flight or wavelength resolution significantly reduces reflection overlap, enabling data collection from large unit-cell axes while also significantly lowering background and increasing the signal-to-noise ratio.

Single-crystal neutron diffraction experiments have historically been limited by the available neutron flux, which has always been orders of magnitude lower than available X-ray fluxes. However, with the development of deuterium labeling techniques and new instruments, it is becoming a more readily used technique. A number of recent review articles (O’Dell *et al.*, 2016 ▸; Blakeley, 2009 ▸; Chen & Unkefer, 2017 ▸; Niimura, 2011 ▸; Blakeley *et al.*, 2015 ▸; Oksanen *et al.*, 2017 ▸) have given a current picture of single-crystal macromolecular neutron crystallography which documents a growing vigorous activity in neutron protein crystallography worldwide. Several instruments such as LADI-III (Blakeley *et al.*, 2010 ▸), IMAGINE (Meilleur *et al.*, 2013 ▸), BIODIFF (Coates *et al.*, 2014 ▸) and IBIX (Tanaka *et al.*, 2010 ▸) are now available for single-crystal macromolecular neutron crystallography, which can collect data on crystal volumes between 0.1 and 1 mm^3^. MaNDi at the Spallation Neutron Source (SNS) is the most recently developed macromolecular neutron diffractometer instrument (Coates *et al.*, 2010 ▸, 2015 ▸). The SNS produces neutrons in discrete pulses 60 times a second. On MaNDi, a series of bandwidth choppers selects the wavelengths of neutrons which will be used in the experiment from within these pulses. These neutrons then interact with and are scattered by the sample into a large array of time-sensitive detectors which surround the sample. MaNDi has a bandwidth of Δλ = 2.16 Å, with neutrons between 2 and 4.16 Å typically being used in macromolecular experiments. As the time of generation for each neutron pulse, the so-called ‘T0’, and the length of MaNDi are well known, the wavelength of the detected neutrons can easily be determined by measuring their time of flight (TOF) (Langan *et al.*, 2008 ▸). This enables the Laue diffraction patterns recorded on MaNDi to be divided into monochromatic slices by sorting the data into discrete TOF ranges, thus massively reducing reflection overlap, decreasing background and thereby increasing the signal-to-noise ratio (Fig. 1 ▸).

Owing to its high TOF resolution (Schultz *et al.*, 2005 ▸), MaNDi is able to collect data from unit cells up to 300 Å (Azadmanesh *et al.*, 2017 ▸) while also collecting data on crystal volumes down to 0.1 mm^3^. However, to move to crystal volumes an order of magnitude smaller for data collection, improved neutron sources and instrumentation are needed. The high brightness of the cold neutrons currently being designed for the second target station (STS) of the SNS at Oak Ridge National Laboratory (ORNL) will be ideally suited for experiments that require focusing optics to enable measurements on smaller samples than is currently possible using the SNS. This capability will enable the study of proteins from which it is challenging to prepare crystals as large as 0.1 mm^3^ in volume. We report here the design of a new macromolecular neutron diffractometer, Ewald, which is designed for collecting data from large unit cells up to 300 Å on edge while also being optimized for crystal volumes of 0.01 mm^3^ and smaller. The small, high-brightness coupled moderators available at the STS combined with recent advances in neutron instrumentation will deliver smaller, more intense neutron beams to the sample position by the utilization of Montel (nested) Kirkpatrick–Baez (KB) neutron supermirrors (Ice, Barabash & Khounsary, 2009 ▸; Ice, Pang *et al.*, 2009 ▸). This will enable Ewald to collect data from smaller crystals than can currently be studied using existing instrumentation.

## 2. Instrument concept   

**Figure 2:**
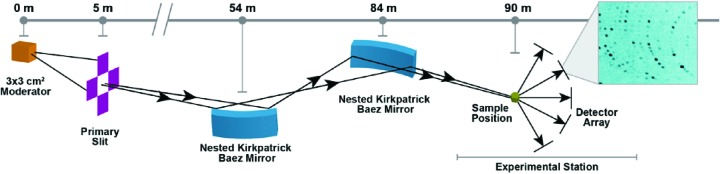
A schematic overview of the neutron optics on Ewald, showing the location of the moderator, primary slit, KB neutron supermirrors, sample position and detector array and a representative diffraction pattern from a single detector module.

The neutron optics system for Ewald (Fig. 2 ▸) is based on a pair of nested elliptical KB focusing neutron supermirrors located at 54 and 84 m from the moderator. These neutron supermirrors are 3 m in length and 15 cm in height and image a neutron slit which will be positioned 5 m from the moderator. The opening of this slit will be de-magnified by a factor of ×30 at the sample position. By varying the size of the slit opening we will be able to adjust the dimensions of the neutron beam at the sample position down to 0.001 mm^2^. This mechanism will enable us to closely match the beam size at the sample position to the dimensions of the crystal to reduce background and increase signal-to-noise ratio. Using this arrangement, we also have no line of sight from the moderator to the sample position, further reducing background while also avoiding any fast neutrons and gammas emitted during neutron production reaching the sample position even in the event of a chopper failure. The horizontal and vertical divergence of the neutron beam at the sample is fixed at 0.38° FWHM across the 1–10 Å wavelength band.

A T0 chopper located at 6.5 m and two bandwidth choppers positioned at 8 and 10 m from the moderator will remove fast neutrons and gammas and select the neutron wavelengths to be used for each experiment. A secondary shutter located at 84.6 m from the moderator will allow the easy change out of samples. At the sample position (90 m) a high-precision goniometer will align and position the crystal into the neutron beam, with neutrons scattered from the sample being detected by a hemispherical array of next-generation high-resolution (HR) SNS Anger camera detectors.

MaNDi views a decoupled hydrogen moderator at the SNS which gives sharp neutron pulses with short emission times (Schultz *et al.*, 2005 ▸) (17.4 µs FWHM at 2 Å), enabling the study of large unit-cell axes up to 300 Å (Schultz *et al.*, 2005 ▸). To ensure the same or better timing resolution for Ewald at the STS, which views a 3 × 3 cm high-brightness coupled moderator, one needs an instrument that is around three times longer to account for the moderator pulse width difference (43.3 µs FWHM at 2 Å). Thus, Ewald has a flight path length of 90 m, three times that of MaNDi. At this length and with the 15 Hz repetition rate of the STS, Ewald will have a bandwidth (Δλ) of 3.0 Å, perfect for neutron protein crystallography as all useful wavelengths (1.5–4.5 Å) can be collected in a single exposure. The key instrument parameters and capabilities of MaNDi and Ewald are given in Table 1 ▸.

## 3. Potential neutron flux gain factors   

Using the *McStas* program (Willendrup *et al.*, 2014 ▸), we have conducted several initial Monte Carlo simulations of Ewald to assess its performance relative to that of MaNDi at the first target station of SNS. On MaNDi (Coates *et al.*, 2010 ▸) with a fixed beam divergence of 0.38° at the sample position, the flux on sample is 1.3 × 10^5^ n s^−1^ mm^−2^ for all neutrons between 2 and 4.16 Å. The higher-brightness coupled moderator available at the STS combined with a KB neutron optics system has enabled us to increase the flux on sample at Ewald to 7.64 × 10^6^ n s^−1^ mm^−2^ for all neutrons between 1.5 and 4.5 Å, with the same fixed beam divergence of 0.38°, giving us a simulated gain factor in flux of ×59.

**Figure 3:**
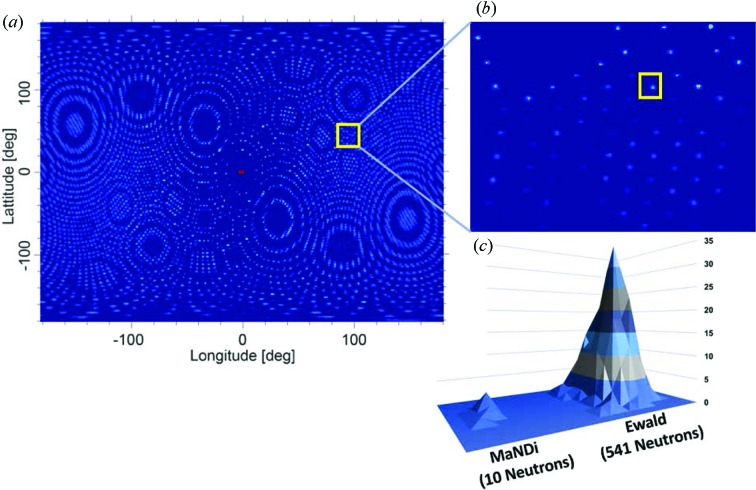
(*a*) Simulated diffraction from Ewald onto a 4π spherical detector located 300 mm from the sample. The large number of Bragg peaks stimulated by such a large unit cell can be visualized. (*b*) Diffraction recorded on a single high-resolution SNS Anger camera at a 2θ angle of 90° is shown for Ewald. (*c*) A close-up of a simulated Bragg peak with *D*_min_ = 2.0 Å is shown for Ewald and MaNDi. The Bragg peak produced from Ewald is composed of 541 neutron counts compared to 10 from MaNDi, highlighting the ability of Ewald to collect meaningful data on crystal volumes of 0.01 mm^3^ and below.

We have simulated the performance of Ewald on a small crystal (0.01 mm^3^) of an inorganic pyrophosphatase (IPPase) using the *McStas* program (Fig. 3 ▸). IPPase is an enzyme that catalyzes the conversion of one mol­ecule of pyrophosphate to two phosphate ions, which is a highly exergonic reaction. This reaction is often coupled to unfavorable biochemical transformations to help drive them to completion. The functionality of this enzyme plays a critical role in lipid synthesis and degradation, bone formation, and DNA synthesis. The protein itself is a hexamer in the asymmetric unit formed from six protein chains, each being composed of 174 amino acids (Hughes *et al.*, 2012 ▸). This large complex is a challenging target for neutrons, and initial data collection at the protein crystallography station instrument located at the LANSCE facility in Los Alamos, NM, USA, required a crystal over 500 times larger in volume (5 mm^3^) for data collection (Hughes *et al.*, 2012 ▸). The specifics of the simulations conducted for Ewald and MaNDi are given in Table 2 ▸. A narrow TOF range corresponding to neutrons between 2.82 and 2.84 Å was used for the Monte Carlo simulations owing to the large number of reflections generated from such a large unit cell.

**Figure 4:**
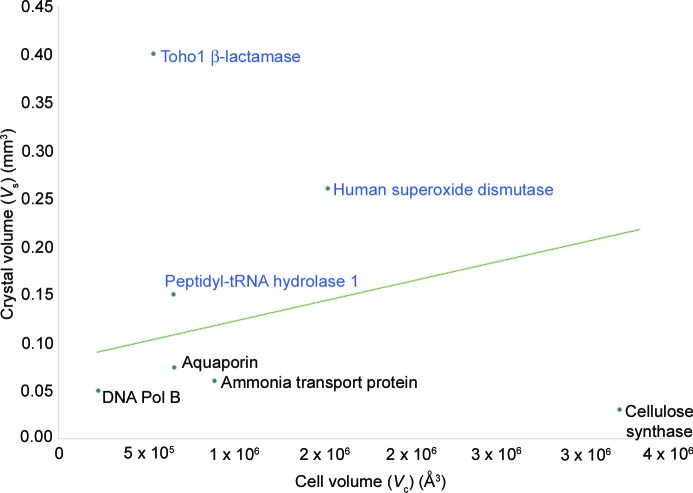
A scatter plot of examples of challenging protein crystals collected on MaNDi (blue text) and several examples of protein crystals that the Ewald instrument can bring into range for neutron crystallography (black text). The green line shows an empirical limit which has been observed on the MaNDi instrument.

The average intensity of a Bragg reflection (*I*) for a particular crystal depends upon the number of unit cells within it. This can easily be calculated as *I* = *V*_s_/*V*_c_, where *V*_s_ is the crystal volume and *V*_c_ is the volume of the unit cell. In Fig. 4 ▸ we have constructed a Wilkinson scatter plot (Habash *et al.*, 1997 ▸) for some of the challenging samples that have been collected on MaNDi so far, along with details of samples that will likely become feasible on the Ewald instrument.

Thus, by reducing the minimum crystal size requirement for a successful data collection, many new types of proteins become amenable to neutron protein crystallography. These include membrane proteins, DNA repair proteins and enzymes of interest in the production of biofuels.

## 4. Dynamical neutron polarization   

**Figure 5:**
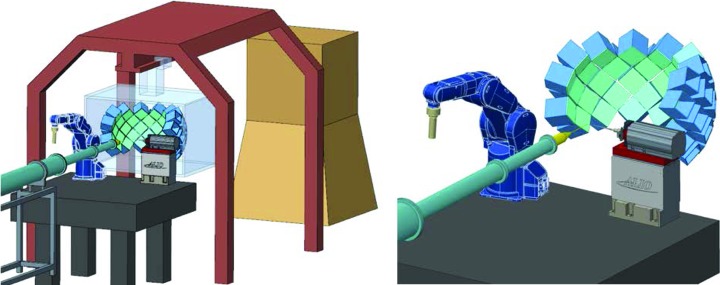
The current model of the Ewald end station is shown. A large detector array composed of 37 HR-SNS Anger cameras is designed to reduce the number of orientations required to collect a complete dataset. A high-resolution goniometer, nitrogen cryostream and robotic sample changer will allow for offsite operation.

Dynamical neutron polarization (DNP) uses a combination of high magnetic fields and low temperature to enhance and manipulate the nuclear polarization in macromolecular crystals, giving the ability to control the neutron cross section (Pierce *et al.*, 2009 ▸, 2010 ▸). *In situ* control of the neutron cross section will significantly enhance the contribution of hydrogen, which accounts for over half the atoms in a typical protein crystal, to the measured signal while simultaneously minimizing the incoherent scattering background. This will potentially reduce the crystal size required for Ewald by a further order of magnitude. Ewald has been flexibly designed to accommodate DNP equipment and sample environments. The end station of the Ewald instrument is initially designed with 37 HR-SNS Anger cameras (Fig. 5 ▸), which are mounted on a hemispherical movable detector array frame that can be easily retracted to allow for the installation of DNP apparatus.

At a sample-to-detector distance of 300 mm, the detector array provides a coverage of 5.3 sr compared to 4.1 sr on the MaNDi instrument. Ewald will also utilize the next generation of SNS Anger cameras, which unlike previous generations are not sensitive to stray magnetic fields, enabling DNP deployment on Ewald. A high-precision goniometer at the sample position aligns crystals into the neutron beam, while a robotic sample changer allows for automated data collection and remote operation of the beamline. Further work on the instrument design will include modeling the effects of gravity on the neutron optics system and improving detector coverage.

## 5. Conclusions   

Current neutron instrumentation is able to collect data on crystals an order of magnitude smaller than previous generations of instrumentation, which typically required crystals greater than 1 mm^3^. To date, 119 neutron protein structures have been deposited in the Protein Data Bank, with the first listed deposition occurring in 1984. However, around 60% of these 119 structures were deposited within the past five years, indicating that this decrease in sample volume requirement has distinctly increased the reach of neutron protein crystallography. The construction of the STS at SNS with its compact brighter moderators will allow the deployment of novel neutron focusing optics which are able to transmit smaller higher-flux neutron beams to the sample position. This combined with the ability to manipulate neutron scattering cross sections with DNP will allow for protein crystals orders of magnitude smaller to be used for data collection. While neutron protein crystallography will always be used for hypothesis-driven research, the construction of Ewald at the STS will at last enable neutron protein crystallography to address many more interesting science questions.

## Figures and Tables

**Table 1:** The key instrument parameters and capabilities of MaNDi and Ewald

Instrument		MaNDi	Ewald
Length (m)		30	90
Bandwidth (Å)		2.16	3.0
Detector coverage (sr)		4.1	5.3
Detector pixel size (mm)		1	0.3
Crystal-to-detector distance (cm)		45	30
Beam size at sample position (mm^2^)		Variable (1–7)	Variable (0.001–1)
Beam divergence at sample (°)		Variable (0.12–0.8)	0.38
Chopper locations (m)		7.2, 8.27, 10.50	6.5, 8, 10

**Table 2:** The specifics of the simulations conducted for Ewald and MaNDi

Instrument		Ewald		MaNDi	
Wavelength center (Å)		2.83		2.83	
Simulated detector type		HR-SNS Anger camera		SNS Anger camera	
Crystal-to-detector distance (mm)		300		450	
Detector pixel size (mm^2^)		0.3		1	
Detector coverage (sr)		5.3		4.0	
Space group		*C*2		*C*2	
*a*, *b*, *c* (Å)		106.10, 95.51, 113.73		106.10, 95.51, 113.73	
α, β, γ (°)		90.00, 98.08, 90.00		90.00, 98.08, 90.00	
Resolution range (Å)		19.68–1.50		19.68–1.50	
Total No. of possible reflections		1 795 238		1 795 238	
Number of atoms (asymmetric unit)		18 613		18 613	
Crystal volume (mm^3^)		0.01		0.01	
Crystal mosaic spread (°)		0.3		0.3	
